# Assessment of long axis shortening with cardiac magnetic resonance imaging. A validation study with different techniques

**DOI:** 10.1186/1532-429X-17-S1-P384

**Published:** 2015-02-03

**Authors:** Johannes Riffel, Malte Maertens, Franziska Rost, Florian Andre, Sorin Giusca, Grigorios Korosoglou, Hugo Katus, Sebastian Buss

**Affiliations:** 1 Cardiology, University of Heidelberg, Heidelberg, Germany

## Background

Assessment of longitudinal function in cardiac magnetic resonance imaging (CMR) is limited to systolic excursion of the mitral annulus (MAPSE) or elaborate strain imaging modalities. The aim of the study was to evaluate long axis shortening (LAS) as a fast assessable and feasible parameter for measurement of longitudinal function in CMR.

## Methods

60 healthy volunteers, 60 patients with dilative cardiomyopathy, 40 patients with AL amyloidosis and 25 patients with hypertrophic cardiomyopathy underwent a CMR examination. 4 different techniques for the measurement of LAS were consecutively evaluated. Two of the techniques (LAS-epi/perp and LAS endo/perp) were assessed by measuring the distance between the epicardium or endocardium of the LV apex and a line connecting the origins of the mitral valve leaflets perpendicularly in end-diastole and end-systole. In the two other methods (LAS-epi/mid and LAS-endo/mid) the distance between the middle of the line connecting the mitral valvue leaflets and the epicardium or endocardium of the apex was measured in end-systole and end-diastole. Values for LAS for all techniques were finally assessed in percent according to the strain formula in 2 and 4 chamber views.

## Results

LAS-epi/mid and LAS-epi/perp displayed the highest sensitivity and specificity in discriminating normal subjects from patients. LAS-epi/mid showed the best correlation with feature tracking derived transmural strain (r=0.85). We therefore identified LAS epi/mid as the most feasible method among the 4 techniques for measurement of long axis function. Assessment of LAS-epi/mid exhibits good intra- and interobserver variabilities

Compared to strain imaging, LAS-epi/mid showed similar values for sensitivity (83.9% vs. 83.9%) and specificity (95.1% vs. 88.5%). ROC analysis revealed comparable values for AUC for LAS-epi/mid (0.95) and mean transmural strain (0.92, p=0.17), respectively.

Moreover, LAS-epi/mid performed significantly better than other parameters of cardiac function such as MAPSE (AUC= 0.87; p< 0.005) and the EF (AUC= 0.83; p=0.0001).

## Conclusions

Measurement of LAS using CMR is feasible for analysis of longitudinal function with high sensitivity and specificity.

## Funding

The study was supported by a grant from the B. Braun Stiftung. H.A.K. was supported by the DZHK (Deutsches Zentrum für Herz-Kreislauf-Forschung - German Centre for Cardiovascular Research).

